# Coexistent Non–Small Cell Carcinoma and Small Cell Carcinoma in a Patient Presenting with Hyponatremia

**DOI:** 10.1155/2018/1718326

**Published:** 2018-02-20

**Authors:** Mitchell D. Ross, Sreeja Biswas Roy, Pradnya D. Patil, Jasmine L. Huang, Nitika Thawani, Ralph Drosten, Tanmay S. Panchabhai

**Affiliations:** ^1^Department of Internal Medicine, St. Joseph's Hospital and Medical Center, Phoenix, AZ, USA; ^2^Department of Hematology and Oncology, Taussig Cancer Institute, Cleveland Clinic, Cleveland, OH, USA; ^3^Norton Thoracic Institute, St. Joseph's Hospital and Medical Center, Phoenix, AZ, USA; ^4^Department of Radiation Oncology, University of Arizona Cancer Center, St. Joseph's Hospital and Medical Center, Phoenix, AZ, USA; ^5^Department of Radiology, St. Joseph's Hospital and Medical Center, Phoenix, AZ, USA

## Abstract

Despite recent advances in screening methods, lung cancer remains the leading cause of cancer-related deaths worldwide. By the time lung cancer becomes symptomatic and patients seek treatment, it is often too advanced for curative measures. Low-dose computed tomography (CT) screening has been shown to reduce mortality in patients at high risk of lung cancer. We present a 66-year-old man with a 50-pack-year smoking history who had a right upper lobe (RUL) pulmonary nodule and left lower lobe (LLL) consolidation on a screening CT. He reported a weight loss of 45 pounds over 3 months, had recently been hospitalized for hyponatremia, and was notably cachectic. A CT of the chest showed a stable LLL mass-like consolidation and a 9 × 21 mm subsolid lesion in the RUL. Navigational bronchoscopy biopsy of the RUL lesion revealed squamous non–small cell lung cancer (NSCLC). Endobronchial ultrasound-guided transbronchial needle aspiration of the LLL lesion revealed small cell lung cancer (SCLC). The final diagnosis was a right-sided Stage I NSCLC (squamous) and a left-sided limited SCLC. The RUL NSCLC was treated with stereotactic radiation; the LLL SCLC was treated with concurrent chemotherapy and radiation. In patients with multiple lung nodules, a diagnosis of synchronous multiple primary lung cancers (MPLCs) is crucial, as inadvertent upstaging of patients with MPLC (to T3 and/or T4 tumors) can lead to erroneous staging, inaccurate prognosis, and improper treatment. Recent advances in the diagnosis of small pulmonary nodules via navigational bronchoscopy and management of these lesions dramatically affect a patient's overall prognosis.

## 1. Introduction

Lung cancer is by far the leading cause of cancer deaths in both men and women, with roughly 222,500 new cases of lung cancer expected to be diagnosed in 2017 alone [1]. The National Lung Screening Trial (NLST) [2], published in 2011, demonstrated that lung cancer screening can detect early-stage lung cancer, thereby decreasing mortality [2]. In addition to detection of early-stage lung cancer, screening programs may incidentally reveal other findings, such as nonmalignant lung nodules and coronary artery disease (based on coronary calcium scoring) [2]. Pulmonary nodules detected during lung cancer screening are managed using the Fleischner Society recommendations [3] or the Lung-RADS™ guidelines [4].

The most common incidental finding is a single primary malignancy, but synchronous multiple primary lung cancers in varied stages are occasionally diagnosed based on lung cancer screening results [5]. However, the approach to such distinct lung nodules is not always straightforward, and appropriate staging based on tissue diagnosis is of paramount importance. In Western societies, the incidence of synchronous primary lung cancers has been reported to be anywhere from 0.2% to 20% [6–10]. Here we report the unique presentation, challenging diagnosis, and successful management of non–small cell lung cancer (NSCLC) and small cell lung cancer (SCLC) coexisting in the same patient.

## 2. Case Presentation

A 66-year-old man with a 50-pack-year smoking history and severe chronic obstructive pulmonary disease (COPD) presented to our clinic with a new right upper lobe (RUL) pulmonary nodule and a chronic left lower lobe (LLL) consolidation on screening computed tomography (CT). His pulmonary function tests (PFTs) showed airflow obstruction with an FEV_1_ of 2.58 L (81% predicted). The LLL consolidation was evident on a chest radiograph 6 months before his current presentation, and the lesion had been biopsied by his local pulmonologist via conventional bronchoscopy with no evidence of malignancy.

**Figure 1:**
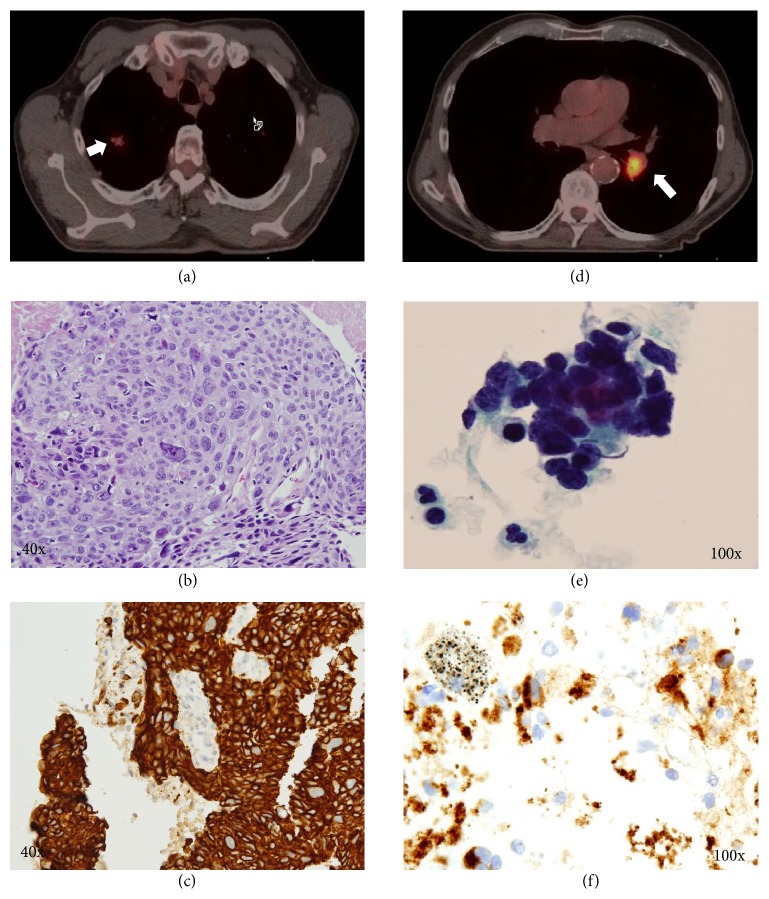
Positron emission tomogram showing mild [11F]-2-fluoro-2-deoxy-D-glucose (FDG) uptake in the right upper lobe lesion (a) (short white arrow), diagnosed as squamous cell carcinoma on biopsy ((b) hematoxylin and eosin, 40x; (c) CK5 immunostain, 40x). (d) A highly FDG-avid left infrahilar mass (long white arrow) that was diagnosed as small cell carcinoma after biopsy analysis ((e) hematoxylin and eosin, 100x; (f) synaptophysin, 100x).

The patient reported a 45-pound weight loss over the course of three months, as well as a recent hospitalization for severe hyponatremia (109 mEq/L). Upon physical examination, the patient was noted to be cachectic, with bilateral decreased air entry. CT of the chest showed a stable LLL consolidation along with a 9 × 21 mm subsolid lesion in the RUL. Navigational bronchoscopy and biopsy of the RUL lesion revealed squamous NSCLC with positive CK5, p63, and TTF-1 markers (Figures 1(a)–1(c)). The LLL consolidation was not sampled during this procedure, as it was located on the contralateral lung, was noted to be stable for 6 months based on a prior chest radiograph, and had been analyzed after a previous bronchoscopy with cytology and transbronchial lung biopsies without evidence of malignancy. A staging positron emission tomogram (PET) done after the navigational bronchoscopy once a malignant diagnosis was confirmed showed FDG avidity in the RUL lesion (SUV 2), along with an FDG-avid left infrahilar mass (SUV 11) within the LLL consolidation (Figure 1(d)). The absence of enlarged or FDG-avid mediastinal adenopathy made the diagnosis of a second primary cancer very likely. Endobronchial ultrasound-guided transbronchial needle aspiration (EBUS-TBNA) of the left infrahilar mass revealed a cluster of cells with minimal cytoplasm and small nuclei with nuclear cytoplasmic molding (Figures 1(d)–1(f)).

Immunohistochemical markers were consistent with an undifferentiated SCLC that was positive for CAM 5.2, TTF-1, CD56, and synaptophysin and negative for p63 and CK5 (Figure 1). The final diagnosis was a right-sided Stage I NSCLC (squamous) and a left-sided limited SCLC. Chemotherapy was initiated with etoposide and cisplatin, along with concurrent radiation for the left-sided SCLC. Given the overall poor survival associated with small cell carcinoma, surgery including segmentectomy was not a therapeutic option for the Stage I right-sided squamous cell carcinoma. Stereotactic body radiation therapy (SBRT) was then initiated for the squamous cell cancer. Despite lower rates of recurrence after segmentectomy compared to SBRT, the likelihood of recurrent SCC driving survival in this patient with concurrent SCLC was low. The patient's hyponatremia has since resolved, and his surveillance PET scan showed him to be recurrence-free at 1 year.

## 3. Discussion

Lung cancer screening has been shown to decrease mortality associated with lung cancer by detecting early-stage lung cancer in high-risk patients [2]. Patients at increased risk for lung cancer include those who are between the ages of 55 and 74 years and who have at least a 30-pack-year smoking history. Lung nodules detected on screening CT can then be risk-stratified based on either Fleischner Society recommendations [3] or the Lung-RADS guidelines [4]. Diagnosis of synchronous multiple primary lung cancers becomes especially important when a satellite lung nodule in the same lobe is staged as a T3 tumor whereas one in an ipsilateral lung lobe is classified as a T4 tumor. Upstaging patients with multiple primary lung cancers (MPLCs) can lead to erroneous staging, inaccurate prognosis, and, ultimately, improper treatment.

Recent advances in the diagnosis of small pulmonary nodules via navigational bronchoscopy and the management of these nodules (either surgically or with SBRT) can significantly affect patients' prognosis, survival, and treatment. To diagnose synchronous MPLCs, physicians must adhere to strict criteria and obtain tissue samples of both lesions. These samples must each be malignant and must arise independently in the lung. Benign nodules, infectious processes, or metastases from extrapulmonary sources must be excluded. Further complicating the diagnostic process is the fact that up to 50% of synchronous MPLCs are composed of multiple adenocarcinomas [11].

The accepted criteria for distinguishing a second primary malignancy from metastasis are

1. different histology from a separate focus,
2. same histology but anatomically distinct lesions without involvement of the mediastinum (N2/N3) and without systemic metastases,
3. same histology but clearly different lesions (so designated based on predominant subtype, cytological features, or different biomarker patterns) [12].

Our patient met the criteria of different histology, anatomically distinct lesions, and different biomarkers. Before biopsy, the absence of mediastinal involvement on his PET scan triggered suspicion for synchronous MPLCs. Any patient with multiple lung nodules without mediastinal involvement should undergo tissue sampling of all lung lesions for definitive diagnosis.

Current recommendations suggest that clinicians initiate a discussion about lung cancer screening with patients who meet certain criteria. Patients aged 55 to 74 years who have at least a 30-pack-year smoking history, who currently smoke, or who have quit within the past 15 years and those who are in relatively good health qualify for screening [13]. The recommended screening modality is annual low-density CT. Lung cancer screening is associated with a 20% relative decrease in lung cancer deaths and a 7% relative reduction in all-cause mortality in these high-risk patients [2].

Our case depicts the insidious development of synchronous primary lung cancer with both NSCLC and SCLC, and with this case come several crucial teaching points. Patients diagnosed with one cancer are at increased risk of a second malignancy, and patients with one pulmonary neoplasm are at increased risk of a second lung tumor [8]. MPLCs are present in just 0.5% of patients diagnosed with lung cancer, and NSCLC and SCLC presenting as synchronous primary tumors are exceedingly rare. Our patient's severe hyponatremia tipped us off to these MPLCs, as severe hyponatremia is a common paraneoplastic syndrome associated with SCLC. Tissue diagnosis for such lesions (without mediastinal involvement) may have a significant impact on an individual's therapy and prognosis. Innovative modalities, including surgical resection of one primary tumor and SBRT for the other, are increasingly common for synchronous early-stage cancers (when both tumors are NSCLC). Therefore, proper diagnosis and staging of all suspicious pulmonary nodules are of paramount importance in the ever-changing, high-stakes field of lung cancer.

## References

[1] Siegel R. L., Miller K. D., Jemal A. (2017). Cancer statistics, 2017. *CA: A Cancer Journal for Clinicians*.

[2] Aberle D. R., Adams A. M., Berg C. D. (2011). Reduced lung-cancer mortality with low-dose computed tomographic screening. *The New England Journal of Medicine*.

[3] MacMahon H., Naidich D. P., Goo J. M. (2017). Guidelines for management of incidental pulmonary nodules detected on CT images: from the Fleischner Society 2017. *Radiology*.

[4] Pinsky P. F., Gierada D. S., Black W. (2015). Performance of lung-RADS in the national lung screening trial: A retrospective assessment. *Annals of Internal Medicine*.

[5] Aziz T. M., Saad R. A., Glasser J., Jilaihawi A. N., Prakash D. (2002). The management of second primary lung cancers. A single centre experience in 15 years. *European Journal of Cardio-Thoracic Surgery*.

[6] Rea F., Zuin A., Callegaro D., Bortolotti L., Guanella G., Sartori F. (2001). Surgical results for multiple primary lung cancers. *European Journal of Cardio-Thoracic Surgery*.

[7] Rostad H., Strand T.-E., Naalsund A., Norstein J. (2008). Resected synchronous primary malignant lung tumors: a population-based study. *The Annals of Thoracic Surgery*.

[8] Stiles B. M., Schulster M., Nasar A. (2015). Characteristics and outcomes of secondary nodules identified on initial computed tomography scan for patients undergoing resection for primary non-small cell lung cancer. *The Journal of Thoracic and Cardiovascular Surgery*.

[9] Tanvetyanon T., Robinson L., Sommers K. E. (2010). Relationship between tumor size and survival among patients with resection of multiple synchronous lung cancers. *Journal of Thoracic Oncology*.

[10] Trousse D., Barlesi F., Loundou A. (2007). Synchronous multiple primary lung cancer: An increasing clinical occurrence requiring multidisciplinary management. *The Journal of Thoracic and Cardiovascular Surgery*.

[11] Bhaskarla A., Tang P. C., Mashtare T. (2010). Analysis of second primary lung cancers in the SEER database. *Journal of Surgical Research*.

[12] Detterbeck F. C., Franklin W. A., Nicholson A. G. (2016). The IASLC lung cancer staging project: background data and proposed criteria to distinguish separate primary lung cancers from metastatic foci in patients with two lung tumors in the forthcoming eighth edition of the TNM classification for lung cancer. *Journal of Thoracic Oncology*.

[13] Wood D. E. (2015). National Comprehensive Cancer Network (NCCN) clinical practice guidelines for lung cancer screening. *Thoracic Surgery Clinics*.

